# Assessing the Influence of Land Use and Land Cover Datasets with Different Points in Time and Levels of Detail on Watershed Modeling in the North River Watershed, China

**DOI:** 10.3390/ijerph10010144

**Published:** 2012-12-27

**Authors:** Jinliang Huang, Pei Zhou, Zengrong Zhou, Yaling Huang

**Affiliations:** 1 Fujian Provincial Key Laboratory of Coastal Ecology and Environmental Studies, Xiamen University, Xiamen 361005, China; 2 College of the Environment and Ecology, Xiamen University, Xiamen 361005, China; E-Mails: peizhou.0419@gmail.com (P.Z.); huangyaling0602@163.com (Y.H.); 3 Department of Environmental Science and Engineering, Huaqiao University, Xiamen 361021, China; E-Mail: zhouzengrong@hqu.edu.cn

**Keywords:** LULC, SWAT, streamflow, loads, sensitivity, watershed modeling

## Abstract

Land use and land cover (LULC) information is an important component influencing watershed modeling with regards to hydrology and water quality in the river basin. In this study, the sensitivity of the Soil and Water Assessment Tool (SWAT) model to LULC datasets with three points in time and three levels of detail was assessed in a coastal subtropical watershed located in Southeast China. The results showed good agreement between observed and simulated values for both monthly and daily streamflow and monthly NH_4_^+^-N and TP loads. Three LULC datasets in 2002, 2007 and 2010 had relatively little influence on simulated monthly and daily streamflow, whereas they exhibited greater effects on simulated monthly NH_4_^+^-N and TP loads. When using the two LULC datasets in 2007 and 2010 compared with that in 2002, the relative differences in predicted monthly NH_4_^+^-N and TP loads were −11.0 to −7.8% and −4.8 to −9.0%, respectively. There were no significant differences in simulated monthly and daily streamflow when using the three LULC datasets with ten, five and three categories. When using LULC datasets from ten categories compared to five and three categories, the relative differences in predicted monthly NH_4_^+^-N and TP loads were −6.6 to −6.5% and −13.3 to −7.3%, respectively. Overall, the sensitivity of the SWAT model to LULC datasets with different points in time and levels of detail was lower in monthly and daily streamflow simulation than in monthly NH_4_^+^-N and TP loads prediction. This research provided helpful insights into the influence of LULC datasets on watershed modeling.

## 1. Introduction

Land use and land cover (LULC) datasets are important for watershed assessment and runoff modeling. Environmental modeling requires accurate LULC datasets to parameterize the physical system being simulated [1]. For diffuse pollution models such as the Soil and Water Assessment Tool (SWAT), AnnAGNPS, AVGWLF and simple equation methods using runoff coefficients or pollutant export coefficient, LULC datasets are critical for assigning parameters related to the hydrology and water quality such as curve number, C and P factors involved in the USLE equation from the relevant models’ manual or literature [2,3,4,5,6]. Whether using simple or complex models, an accurate LULC dataset with an appropriate spatial or temporal resolution and level of detail is paramount for reliable predictions. Undoubtedly, understanding the sensitivity of watershed modeling to different LULC dataset sources is an important step in the selection of an appropriate LULC dataset for a particular application.

Numerous studies illustrate the application of LULC datasets in watershed modeling through developing the model approach to simulate the pattern of land use changes and its consequence in the water environment. Land change models were firstly used to develop land use change scenarios and characterize LULC dynamics [2,7,8]. Watershed models were then applied to evaluate the associated impacts on hydrology and water quality [2,7,9,10,11]. 

The physically based, distributed model, SWAT is considered as one of the most suitable models for predicting impacts of land use on water, and nutrition yield in watersheds with varying land use and management conditions [12,13]. Using the SWAT model, some authors evaluate the influence of LULC datasets on runoff, and water quality by developing different artificial land use scenarios with resultant potential environmental consequences [3,14]. Some studies also focus on the sensitivity and uncertainty of the analysis for watershed modeling using SWAT [15,16,17]. However, few studies have evaluated the sensitivity of SWAT simulation to the accuracy of LULC datasets, and this prevents watershed modeling efforts being potential appropriate applications for watershed assessment and management. 

The Jiulong River Basin (JRB) is a medium-sized subtropical coastal watershed located in Southeast China that plays an important role in the surrounding region’s economic and ecological health [18]. However, there is still no clear watershed assessment and modeling in the JRB. The objectives of this study are: (1) to test the applicability of the SWAT model in a coastal subtropical watershed of China, and (2) to explore the relative influence of LULC datasets with different points in time and levels of detail on watershed model simulation in the largest watershed of the JRB. 

## 2. Material and Methods

### 2.1. Study Area

**Figure 1:**
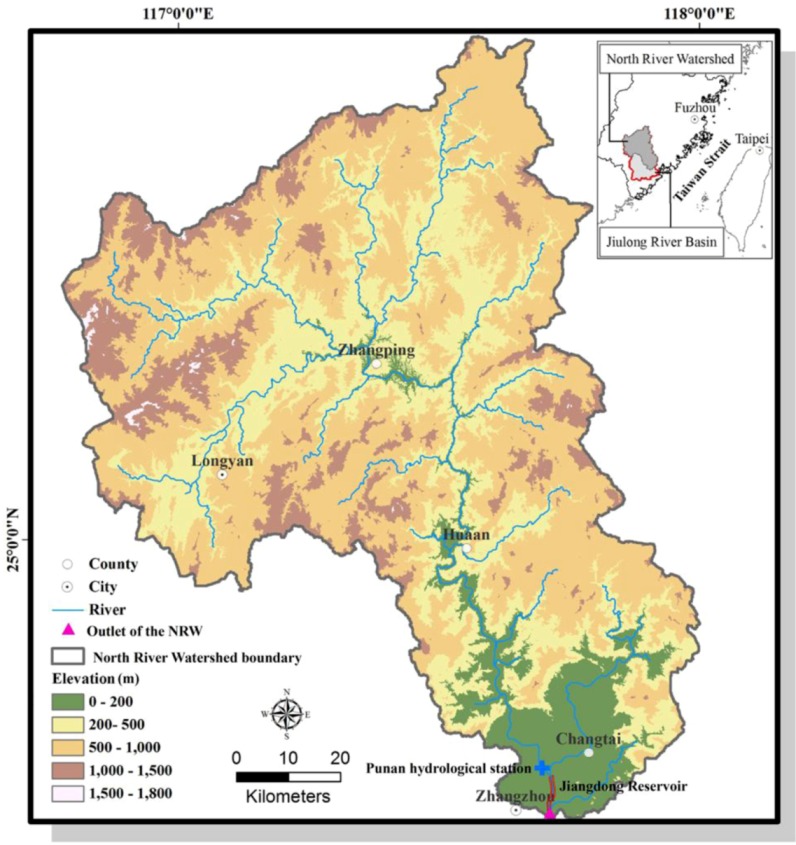
Location of North River watershed.

The North River Watershed (NRW, Figure 1), the largest watershed of the JRB, covers approximately 10,000 km^2^ on the eastern coast of Southeast China (from 116°46′55″E to 118°02′17″E and from 24°31′0.7″N to 25°53′38″N). Approximately 10 million residents from Xiamen, Zhangzhou and Longyan use the North River as their source of water for residential, industrial and agricultural uses. Algal blooms occurred in the Jiangdong Reservoir on the North River over the period from January to February 2009, reflecting the deteriorating water quality situation and the critical need for watershed assessment and management. 

### 2.2. Land Use Classification

Landsat Thematic Mapper (TM) satellite imagery from 2007 and 2010 with a 25 m resolution, and an ETM+ image of 2002 with a 30 m resolution, were used to create land use classifications for each of these three years. After using a geo-referencing procedure with an image-to-image registration method, all the images were re-sampled to a 30 m resolution. The land categories were generated using a combination of unsupervised classification and spatial reclassification based on manual on-screen digitization. Firstly, we identified the threshold values for the infrared (TM3) band and mid-infrared (TM5) band of TM/ETM+ images so as to extract the water, impervious surface area (ISA), and forest spectra, respectively. Then we classified each isolated image by unsupervised classification. The water, ISA and forest spectrum images were separated into 40, 60 and 150 classes, respectively. The images were finally merged into ten classes, namely forest, agriculture, barren, high density residential area, low density residential area, orchard, reservoir, industrial land, transportation and water. Two aggregation steps were performed to investigate the relative differences in simulation outputs using two LULC datasets with different levels of detail. Firstly, high density residential areas, low density residential areas, industrial land and transportation were merged into a category called “Built-up”. Agriculture and orchard were merged into a new category called “Agriculture”. Water and reservoir were merged into a new category called “Water”. Thus we had a new LULC classification system with five LULC categories, namely Built-up, Agriculture, Forest, Barren and Water. Secondly, based on the LULC datasets with five categories, Forest, Barren, and Water were further merged into a new category called “Natural”, which finally resulted in a new LULC dataset with three categories, that is, Natural, Agriculture and Built-up. It should be noted that all the aggregations are based on the consideration that specific land use categories can reflect specific underlying human activities.

Extensive field surveys were conducted during 11–14 August 2009 to associate the ground information of a specific land category with its imaging characteristics. More than 300 digital photos and GPS points were taken for different land categories. We used this information and some obvious spectral signatures to identify 256 places where a land category persisted over time. We then used those places to generate ground reference information to perform accuracy assessment for the classified maps for all three points in time. Compared with the method used [18], this improved classified method shows preferable accuracy and the overall classification accuracy of three imageries in 2002, 2007 and 2010 are 82.3, 83.2 and 83.7%, respectively.

### 2.3. Parameterization, Calibration and Verification of the SWAT Model

SWAT is a physically based, continuously distributed model, developed by the Agricultural Research Service of the United States Department of Agriculture for simulating the impact of land management practices on water, sediment and agrochemical yields in large watersheds with varying soils, land use and agricultural conditions over extended time periods [19]. More details about SWAT are available from the documents by Neitsch *et al*. [20,21]. In our study, we used the SWAT2000 version.

**Figure 2:**
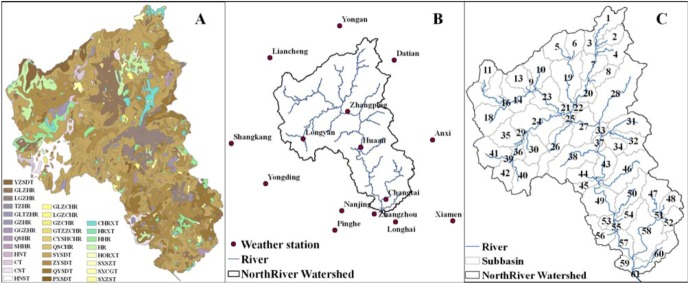
Soil maps (**A**), weather station locations (**B**) and watershed delineation (**C**) in the NRW.

Table 1 gives information for the major input data for SWAT. It should be noted that a part of the soil database related to soil property in SWAT was estimated with SPAW Hydrology or the default value in SWAT and the quadratic interpolation method was used to transform soil data into the American version with MATLAB based on genetic classification (Figure 2(A)) [22,23]. Meteorological data were obtained from 15 weather stations in the NRW (Figure 2(B)). The watershed was discretized into 61 sub-basins (Hydrological Response Units) with dominant land use and soil classification (Figure 2(C)). 

**Table 1:** Description of major input data for SWAT parameterization.

Data	Data Format	Data Source
DEM	Grid (cell size 30 × 30 m)	DEMs from Fujian Provincial Geomatics Center
Land use map	Grid (cell size 30 × 30 m)	TM/ETM+ images classification
Soil map	Vector map (Shapefile)	Soil surveys in Fujian province
Meteorological data	Table (.dbf and text)	Climate stations

The performance of the model in simulating streamflow and nutrients was evaluated using Nash–Sutcliffe efficiency (E_NS_) [24] and the coefficient of determination (R^2^) [25]. The equations used were as follows:

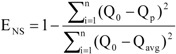
(1)

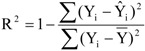
(2)
where Q_0_ and Q_p_ are the observed and simulated data, respectively, Q_avg_ is the average of the observed data and n is the total number of data records. Y_i_ denotes the value of the *i*th dependent variable, 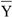 is the mean of the dependent variable and Ŷ_i_ is the *i*th fitted value. 

E_NS_ is widely used to evaluate model performance and normally ranges from 0.0 to 1.0. Its optimal value is 1.0, which is the highest possible value indicating best fit. R^2^ normally ranges from 0.0 to 1.0 and the fitting effect is better as R^2^ approaches 1.0.

SWAT was calibrated and validated using meteorological data and streamflow data gathered from 1 January 2000 until 31 December 2003 and 1 January 2004 until 31 December 2007 at the 15 weather stations and at the outlet of the NRW, respectively. As for the water quality modeling, ammonia nitrogen (NH_4_^+^-N) and total phosphorus (TP) data from January 2001 to December 2003 were used for the calibration effort, and the validation period was from 1 January 2004 until 31 December 2007. 

### 2.4. Scenarios Designed to Evaluate the Influence of LULC Datasets on Watershed Modeling

#### 2.4.1. An Investigation of the Relative Impact of an Old LULC Dataset (the 2002 LULC Dataset Used for Calibration and Validation) *versus* Two Later LULC Datasets (LULC Datasets in 2007 and 2010)

After calibration and validation, SWAT was further applied in this study to predict the monthly and daily streamflow, and monthly NH_4_^+^-N and TP loads in 2010 in the NRW. It should be noted that the LULC dataset for calibration and validation processes was the LULC in 2002. In this scenario, all the input parameters were kept the same, except for the LULC datasets. Namely, we replaced LULC datasets for 2002 with the LULC datasets for 2007 and 2010. The three cases all used the same meteorological data in 2010. Therefore, the results can reflect the impact of LULC datasets with different points in time on streamflow, NH_4_^+^-N and TP load predictions. 

#### 2.4.2. An Investigation of the Relative Impact of Finer Classification *versus* Coarser Classification

The LULC dataset for calibration and validation process was the 2002 LULC dataset with ten categories. In this scenario, we kept all the input parameters the same with exception of the LULC dataset. We developed two additional LULC datasets with three and five categories. Therefore we had three LULC datasets in 2002 with three, five and ten categories, respectively. The three cases all used the same meteorological data for 2010. As a result, we could examine the relative differences in predicted streamflow, NH_4_^+^-N and TP loads in 2010 resulting from LULC datasets with different levels of detail. 

## 3. Results and Discussion

### 3.1. Detection of Land Use and Land Cover Change Over Time

**Figure 3:**
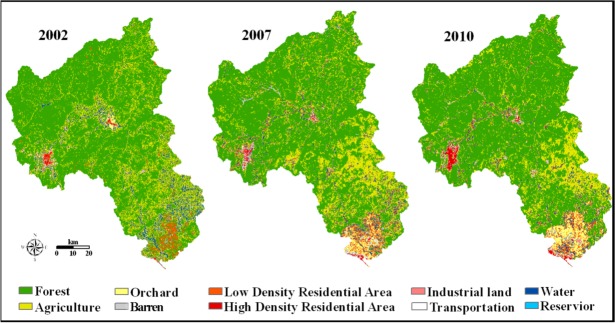
Land use maps in 2002, 2007 and 2010 in the North River Watershed.

Figure 3 shows the maps of ten categories of land use for 2002, 2007 and 2010. The major land use types in the NRW were forest and agriculture, accounting for 71–78% and 16–25%, respectively. Forest increased by 3.4 and 1.9 % for the two periods 2002–2007 and 2007–2010, respectively. Built-up, which was combined from high density residential area, low density residential area, industrial land and transportation as mentioned in subsection 2.2 also increased by 1.3 and 1.2 % for these two intervals. Comparatively, Agriculture decreased by 5.8 and 3.2 % over these two intervals. Water and Barren increased and then decreased during the study period (Table 2). 

**Table 2:** Land use structure for three points in time and three levels of detail in the North River Watershed as a percentage of the total watershed (%).

10 categories	Forest	Agriculture	Water	Orchard		Reservoir		Barren	Industrial land	HDRA *	LDRA *	Transportation
2002	71.81	14.96	1.05	10.48		0.25		0.05	0.01	0.52	0.80	0.07
2007	75.26	15.71	1.51	3.96		0.16		0.66	0.21	0.73	1.60	0.20
2010	78.15	12.15	0.78	4.34		0.18		0.46	0.46	1.42	1.82	0.24
**5 categories**	**Forest**		**Agriculture**				**Water**		**Barren**		**Built-up**	
2002	71.81		25.44				1.30		0.05		1.40	
2007	75.26		19.67				1.67		0.66		2.74	
2010	78.15		16.49				0.96		0.46		3.94	
**3 categories**	**Natural**				**Agriculture**				**Built-up**			
2002	73.16				25.44				1.40			
2007	77.59				19.67				2.74			
2010	79.57				16.49				3.94			

* HDRA and LDRA mean high density residential area and low density residential area, respectively.

### 3.2. Calibration and Validation Results

Table 3 gives a summary of the statistics for calibration and validation. Simulated and observed streamflow matched well in the calibration process for monthly and daily streamflow with E_NS_ = 0.86 and 0.85, respectively, as well as in the validation process with E_NS_ = 0.86 and 0.64 for monthly and daily streamflow, respectively (Table 3). R^2^ for monthly streamflow simulation in calibration and validation was 0.89 and 0.95, respectively, indicating a good linear relationship between simulated and observed data. Comparatively, R^2^ for daily streamflow simulation in calibration and validation was relatively lower, namely, 0.65 and 0.64, respectively. The standard deviations (SDs) of observed values were bigger than those of simulated values, indicating actual streamflow variation was higher.

**Table 3:** Evaluation of monthly and daily streamflow simulation.

	Monthly Streamflow (m^3^/s)				Daily Streamflow (m^3^/s)			
	Calibration		Validation		Calibration		Validation	
	Observed	Simulated	Observed	Simulated	Observed	Simulated	Observed	Simulated
Mean	255.7	222.9	255.7	222.9	256.4	223.3	266.9	272.1
SD *	194.9	176.3	246.0	227.5	314.3	254.4	314.3	254.4
Sample numbers	48	48	48	48	1,461	1,461	1,461	1,461
E_NS_	0.86		0.86		0.64		0.60	
R^2^	0.89		0.95		0.65		0.64	

* SD stands for standard deviation.

Prediction of monthly NH_4_^+^-N and TP loads were both acceptable in the calibration process, with E_NS_ values of 0.69 and 0.56, respectively. In the validation process, the simulated and observed values of monthly NH_4_^+^-N and TP also fitted marginally with E_NS_ = 0.57 and 0.49 (Table 4). Meanwhile, R^2^ for NH_4_^+^-N and TP simulation in the calibration process was 0.71 and 0.90, respectively. R^2^ for NH_4_^+^-N and TP in the simulation in validation processes was 0.61 and 0.63, respectively. 

**Table 4:** Evaluation of monthly NH_4_^+^-N and TP simulation.

	Monthly NH_4_^+^-N Load		Monthly TP Load	
	Calibration	Validation	Calibration	Validation
E_NS_	0.69	0.57	0.56	0.49
R^2^	0.71	0.61	0.90	0.63

The results demonstrated that the SWAT, when calibrated, could provide good estimates of monthly and daily streamflow and monthly NH_4_^+^-N and TP loads. Overall, the SWAT performed better in simulating monthly and daily streamflow than monthly NH_4_^+^-N and TP loads.

### 3.3. Influence of LULC Datasets with Different Points in Time on Watershed Modeling

There were no significant differences in predicted monthly streamflow and daily streamflow when using LULC datasets with three points in time, namely, 2002 (02LU), 2007 (07LU) and 2010 (10LU) (Table 5), indicating that the sensitivity of SWAT modeling of LULC datasets with different points in time was low in terms of streamflow simulation. 

This phenomenon might be attributed to the fact that the study area had not undergone significant land use change over the period 2002–2010 and was also likely due to the comprehensive influence of land use and land cover changes. In this study, Forest increased from 71.8 to 78.2% and Built-up increased from 1.4 to 3.9% over the period 2002–2010 (Table 2). Forest increases may have considerably reduced runoff [11], while Built-up increased at the expense of agricultural land and so would lead to less infiltration for more ISAs and a consequently higher runoff amount [26,27]. 

**Table 5:** Comparison of streamflow, NH_4_^+^-N, and TP simulations under the three points in time.

Land Use Type	Streamflow (m^3^/s)			NH_4_^+^-N Load (×10^3^ kg N)			TP Load (×10^3^ kg P)		
	02LU	07LU	10LU	02LU	07LU	10LU	02LU	07LU	10LU
Monthly mean	301.65	299.79	302.34	569.49	506.71	525.14	881.72	839.23	802.22
Changed amount	-	−1.86	0.69	-	−62.78	−44.35	-	−42.49	−79.50
Monthly Changed percentage (%)	-	−0.62	0.23	-	−11.02	−7.79	-	−4.82	−9.02
Daily mean	301.12	299.27	301.79	18.72	16.66	17.26	28.99	27.59	26.37
Daily Changed amount	-	−1.85	0.67	-	−2.06	−1.46	-	−1.40	−2.62
Daily Changed percentage (%)	-	−0.61	0.22	-	−11.00	−7.80	-	−4.83	−9.04

Compared to the streamflow simulation, LULC datasets with different points in time had greater effects on NH_4_^+^-N and TP load simulation, as shown in Table 5. When using the LULC datasets for 2007 and 2010 to compare with that in 2002, the relative differences in predicted monthly NH_4_^+^-N and TP loads were −11.0 to −7.8 % and −4.8 to −9.0 %, respectively. 

Many factors influence nutrients in rivers, including weather, rainfall, catchment hydrology, soils, land use practices, biogeochemical and point sources [28]. The linkage between land use and land cover change and water quality is well documented throughout the world [29,30,31]. Agricultural land is a well known source for nutrients in rivers [32,33]. In our study, the tendency of the TP loads simulated using LULC datasets with three points in time corresponded well with the dynamics of agricultural change over time. The simulated TP load decreased as agriculture shrunk over time (Table 2 and Table 5). Therefore, we can conclude that agricultural land is an important source of the TP load in the NRW. 

In this study, we found that the sensitivity of watershed modeling to LULC datasets with different points in time was lower in terms of streamflow simulation than in NH_4_^+^-N and TP load prediction, which was similar to earlier findings [11], where land use changes were seen to have a relatively minimal effect on runoff and sediment yield whereas they demonstrate a more considerable effect on the pollutant loads. 

### 3.4. Sensitivity of Watershed Modeling to LULC Datasets with Different Levels of Detail

**Figure 4:**
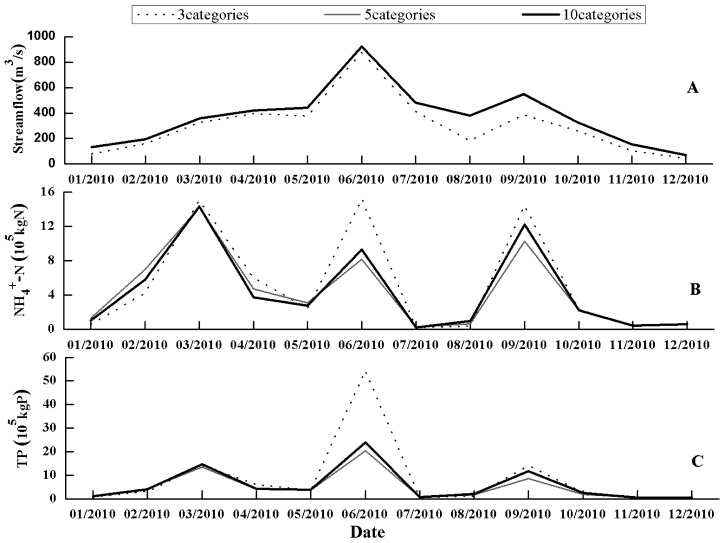
Comparisons among monthly streamflow (**A**), NH_4_^+^-N (**B**) and TP (**C**) loads predicted when using LULC datasets with three levels of detail.

There were little differences in simulated streamflow using the three LULC datasets with ten, five and three categories. In contrast, significant differences in simulated monthly NH_4_^+^-N and TP loads were exhibited when using these three LULC datasets with different levels of detail. When comparing LULC datasets from ten categories to those with five and three categories, the relative differences in predicted monthly NH_4_^+^-N and TP loads were −6.6 to −6.5 % and −13.3 to −7.3 %, respectively (Table 6 and Figure 4). 

The mean values of monthly and daily NH_4_^+^-N and TP loads simulated were lower when using LULC datasets with three and five categories, compared to the simulation results using LULC datasets with ten categories (Table 6). Aggregation can reduce potential map errors [34], while it may result in a considerable loss of information [16]. Therefore, it is understandable that an aggregation procedure, represented by more coarsely classified LULC datasets, resulted in lower mean values of monthly and daily NH_4_^+^-N and TP loads simulated. However, such tendency showed somewhat seasonal variations. As shown in Figure 4, monthly NH_4_^+^-N and TP loads on June 2010 and September simulated using LULC data with three categories was significantly higher than those using LULC data with five and ten categories. 

**Table 6:** Comparison of simulation output with regards to streamflow, NH_4_^+^-N and TP loads when using three LULC datasets with different levels of classification.

	Streamflow (m^3^/s)			NH_4_^+^-N Load (× 10^3^ kg N)			TP Load (× 10^3^ kg P)		
LULC Categories	3	5	10	3	5	10	3	5	10
Monthly mean	300.98	302.26	301.65	532.74	531.95	569.49	817.06	764.32	881.72
Changed amount	−0.67	0.61	-	−36.75	−37.54	-	−64.66	−117.40	
Monthly Changed percentage (%)	−0.22	0.20	-	−6.45	−6.59	-	−7.33	−13.31	
Daily mean	300.42	301.66	301.12	17.51	17.49	18.72	26.86	25.13	28.99
Daily Changed amount	−0.70	0.54	-	−1.21	−1.23	-	−2.13	−3.86	-
Daily Changed percentage (%)	−0.23	0.18	-	−6.46	−6.57	-	−7.35	−13.31	-

Mean values of monthly and daily NH_4_^+^-N and TP loads simulated using LULC data with five categories were lower than those using LULC data with three categories. This might have been caused by the different operations in the SWAT due to the aggregation effects of land use categories. In this study, when using LULC datasets with three categories in the SWAT model, we merged Forest, Barren and Water into “Natural”. Given that Forest has the typical characteristics of “Natural” because of the largest proportion of “Natural” and relatively less anthropogenic disturbance, the new category “Natural” was treated as Forest in the SWAT model. This process can be regarded as afforestation and may reduce streamflow as the higher water holding and conservation properties and evapotranspiration ability of forest [35,36,37]. Therefore, monthly and daily streamflow predicted when using LULC datasets with three categories was a little lower than the simulated results using LULC data with five categories. 

The categories high density residential area, low density residential area, industrial land and transportation were summarized as Built-up for LULC datasets with five and three categories, which was represented by a high density residential area in the SWAT model. Such similar operations may overestimate the role of ISA in urban areas, which could result in the higher values of the NH_4_^+^-N and TP loads simulated when using LULC datasets with ten categories, compared to the NH_4_^+^-N and TP loads simulated using LULC datasets with three and five categories.

Streamflow may increase with the finer classified LULC datasets [38]. However, a watershed modeling analysis of urban catchments based on the SWMM model resulted in an opposite observation that using LULC datasets with coarser spatial resolution and a lower level of classification produces a higher runoff volume and TSS prediction [1]. Comparing the LULC datasets with different levels of detail, there were no significant differences in monthly and daily streamflow predicted while coarser LULC datasets generally predicted lower monthly NH_4_^+^-N and TP loads in this study. The underestimation of NH_4_^+^-N and TP loads with the coarser LULC classification might lead to ignoring a water pollution emergency. Given that diffuse pollution sources and control measures are directly linked to land use, as well as the wide application of environment models for decision making, LULC datasets with different points in time and levels of details should be considered seriously for appropriate watershed assessment and management. 

In this study, we developed two scenarios and used SWAT model which was calibrated and verified to evaluate the relative influence of different LULC datasets on watershed modeling. The simulation results didn’t show significant difference using LULC datasets with different points in time and levels of detail, especially for the streamflow simulation. On the one hand, LULC datasets maybe had little impact because there was little change in the LULC conditions over the study period. On the other hand, the specific operations regarding assigning parameter values to the combined category in the SWAT model system may influence the simulation results. In the next agenda, we need to improve the scenarios development for further model’s applications such as evaluating BMP’s implementation and assessing the effect of dam construction on water quantity and water quality. LULC data issue such as temporal mismatch of data, errors in LULC classification needs to be recognized when exploring the influence of LULC datasets on watershed modeling, which can made the data uncertainty propagated. 

## 4. Conclusions

Understanding the sensitivity of watershed modeling to different LULC dataset sources is an important step in the selection of an appropriate LULC dataset for a particular application. In this study, the sensitivity of the SWAT model to LULC datasets with different points in time and levels of detail was assessed in a coastal subtropical watershed located in Southeast China. The good agreement between observed and simulated values for both monthly and daily streamflow and monthly NH_4_^+^-N and TP loads proved that the SWAT model could provide good estimates of monthly and daily streamflow and monthly NH_4_^+^-N and TP loads. The LULC datasets with three points in time had relatively little impact on monthly and daily streamflow, whereas they exhibited greater effects on NH_4_^+^-N and TP loads. When using two LULC datasets in 2007 and 2010 compared with that in 2002, the relative differences in predicted monthly NH_4_^+^-N and TP loads were −11.0 to −7.8% and −4.8 to −9.0%, respectively. LULC datasets produced little impact on simulation results may be partly due to no significant change in the LULC conditions. There were little differences in simulated monthly and daily streamflow when using LULC datasets with ten, five and three categories. When comparing the LULC datasets from ten categories to five and three categories, the relative differences in predicted monthly NH_4_^+^-N and TP loads were −6.6 to −6.5% and −13.3 to −7.3%, respectively. The specific operations regarding assigning parameter values to the combined category will greatly influence the simulation results. Overall, the sensitivity of the SWAT model to LULC datasets with different points in time and level of details was lower in monthly and daily streamflow simulation than in monthly NH_4_^+^-N and TP loads prediction. The findings of this study provided implications for potentially appropriate applications of the SWAT model for watershed assessment and management.
